# Harmful Consequences of Proton Pump Inhibitors on Male Fertility: An Evidence from Subchronic Toxicity Study of Esomeprazole and Lansoprazole in Wistar Rats

**DOI:** 10.1155/2022/4479261

**Published:** 2022-04-28

**Authors:** Namra Mumtaz, Muhammad Furqan Akhtar, Ammara Saleem, Amjad Riaz

**Affiliations:** ^1^Riphah Institute of Pharmaceutical Sciences, Riphah International University, Lahore Campus, Lahore, Pakistan; ^2^Department of Pharmacology, Faculty of Pharmaceutical Sciences, Government College University Faisalabad, Faisalabad, Pakistan; ^3^Department of Thriogenology, University of Veterinary and Animal Science, Lahore, Pakistan

## Abstract

Proton pump inhibitors (PPIs) are frequently prescribed as gastric acid-suppressing agents. Nevertheless, there is limited evidence supporting the risk of detrimental effects of PPIs on male fertility. The purpose of the current study was to evaluate the effect of subchronic use of proton pump inhibitors on male fertility. Seventy adult male Wistar rats were assigned into seven groups. The normal control group orally received solvent only. Groups 2, 3, and 4 were orally given esomeprazole while groups 5, 6, and 7 received lansoprazole at 2.5, 5, and 10 mg/kg/day, respectively. After 45 days of treatment, blood samples, epididymis, and testis were collected. Sperm count, motility, and morphology were determined. The level of hormones such as testosterone, follicle-stimulating hormone (FSH), and luteinizing hormone (LH) and oxidative status of testis tissue, such as superoxide dismutase, catalase, reduced glutathione, malondialdehyde (MDA), and nitric oxide (NO) were estimated. Results demonstrated a significant decline in sperm count, motility, morphology, testosterone, and catalase at 10 mg/kg/day and GSH at 2.5 mg/kg/day. A significant increase in FSH, LH, and MDA at 10 mg/kg/day and NO at 2.5 mg/kg/day was found as compared to the control group. The pathological alterations specifically dilation of Leydig cells, vacuolization, and degeneration of the seminiferous tubules were also evident. It is concluded that PPIs had caused male reproductive toxicity in Wistar rats due to altered levels of hormones such as testosterone, FSH, and LH, elevated levels of NO, and oxidative stress.

## 1. Introduction

Approximately 15% of couples of reproductive age are globally affected by infertility [1]. Although the etiology of 50% of cases of male infertility remains idiopathic, the major underlying causes of male infertility are impaired semen quality, oxidative stress-mediated sperm damage, endocrine disorders, congenital dysplasia, sexually transmitted infections, varicocele, testicular dysfunction, immune problems, genetic, lifestyle and environmental factors and certain medications such as antidepressants, antiepileptics, calcium channel blockers, *α*-blockers, antibiotics, and antiretroviral therapy [2].

The most significant parameters of sperm quality are sperm motility, sperm count (millions/ml), and sperm morphology [3]. Abnormalities contributing to male infertility include asthenospermia i.e., poor sperm motility, oligospermia, and teratospermia, i.e., irregular sperm morphology [4, 5]. Numerous studies showed that any developmental defect in the flagellum, alteration of seminal pH, abnormalities of sperm protein, and lack of acrosome formation had been associated with asthenospermia [6]. Additionally, an excess of seminal reactive oxygen species (ROS), potential genetic mutations, and a high intake of dietary fatty acids also contribute to reduced sperm motility, thereby causing male infertility [7, 8]. In addition to overproduction of ROS, failure of head and tail attachment, inadequate acrosome formation, and alterations in sperm cytoskeleton promote the formation of morphologically abnormal sperms [9, 10]. In males, hypothalamus-pituitary-gonadal (HPG) axis regulates reproductive hormones, including gonadotropin-releasing hormone (GnRH), follicle-stimulating hormone (FSH), luteinizing hormone (LH), and testosterone. In males, LH is responsible to stimulate Leydig cells, which consequently produce testosterone. In contrast, FSH exerts its actions on the Sertoli cells and seminiferous tubules and plays an important role in the regulation of spermatogenesis [11]. The deficiency of GnRH in men resulted in hypogonadism characterized by a lack of testosterone and impaired spermatogenesis. Several studies revealed that Klinefelter's syndrome, Kallmann syndrome, Y-chromosome microdeletions, testicular trauma, hyperprolactinemia, certain medications such as ketoconazole and opioids, chemotherapy, and radiation are intimately related to the male hypogonadism [12].

Elevated level of ROS has a drastic effect on human spermatozoa and a positive correlation with male infertility. About 30–80% of male infertility issues are associated with pathological levels of ROS. Studies suggested that the structural and functional strength of the sperm membrane were particularly impaired by elevated levels of ROS resulting in increased sperm membrane permeability. ROS also has a direct detrimental effect on sperm DNA and morphology [13]. Additionally, excessive reactive nitrogen species (RNS) such as nitric oxide (NO) have been implicated in inducing testicular dysfunction, abnormal sperm parameters, and reduced gonadotropins secretion [14]. The exact mechanism of oxidative stress-mediated decline in sperm parameters is still unknown but is primarily attributed to lipid peroxidation followed by the production of malondialdehyde (MDA) and DNA fragmentation [15].

Proton pump inhibitors (PPIs) are broadly used medications over the past several decades. Currently, PPIs are available for the management of gastric and duodenal ulcers, erosive esophagitis, gastroesophageal reflux disorders (GERD), as prophylactically for nonsteroidal anti-inflammatory drugs (NSAIDs) mediated bleeding, *Helicobacter pylori* infection and for hypersecretory conditions such as Zollinger–Ellison syndrome. These agents act primarily on hydrogen/potassium ATPase (H^+^/K^+^- ATPase) pump in the gastric parietal cells, resulting in reduced production and secretion of gastric acid [16]. Several studies suggested an association between long-term use of PPIs and potential adverse effects, including iron and vitamin B12 deficiency, hypomagnesemia, risk of bone fracture, particularly hip fracture, *Clostridium difficile* infections, cognitive impairment, and dementia in elder patients [17]. Other studies commented that PPIs can substantially affect sperm quality parameters including sperm count, sperm motility, sperm viability, and capacitation which can lead to male infertility [18].

The deleterious effects of the most widely used PPIs such as esomeprazole and lansoprazole on male reproductive health have been poorly investigated. This inadequacy of data suggests the need for an enhanced investigation of male fertility considering the association between PPIs and male reproductive function. Thus, the current study was carried out to investigate the reproductive toxicity of subchronic exposure to esomeprazole and lansoprazole in male rats and to assess the possible underlying mechanisms of reproductive toxicity associated with esomeprazole and lansoprazole.

## 2. Materials and Methods

### 2.1. Drugs and Chemicals

Carboxymethyl cellulose (CMC), sodium bicarbonate (NaHCO_3_), hydrogen peroxide (H_2_O_2_), chloroform (CHCl_3_), copper sulphate (CuSO_4_), sodium chloride (NaCl), potassium phosphate dibasic (K_2_HPO_4_), potassium dihydrogen phosphate, sodium carbonate, trichloroacetic acid (TCA), dithiobis nitrobenzoic acid (DTNB), thiobarbituric acid (TBA), naphthylethylene diamine dihydrochloride (NEDD), phosphoric acid, sodium potassium tartrate, pyrogallol, formalin, eosin, nigrosin, and methanol were obtained from Sigma-Aldrich Co., USA. Esomeprazole and lansoprazole were acquired from Wuhan Kailun Chemical limited and DMS chemical pharmaceutical limited, China, respectively.

### 2.2. Experimental Animals

Healthy seventy Wistar male rats, 10–12 weeks old, weighing 155 ± 20 g, were acquired and kept under standard laboratory conditions in the animal house at a room temperature (25 ± 2°C) and humidity of 50–70% with proper ventilation with 12 h light and dark cycle. The animals were given a standard rodent pellet diet and water ad libitum. The animal experiments were conducted in accordance with regulations of the University Research Ethical Committee, REC/RIPS-LHR/2017047.

### 2.3. Experimental Design

Male Wistar rats were allocated into seven groups with each group having ten rats (*n* = 10). The control group received 1 ml/animal/day of 0.5% w/v CMC. Groups 2, 3, and 4 were given esomeprazole treatment at 2.5, 5, and 10 mg/kg/day, respectively, whereas Groups 5, 6, and 7 were given lansoprazole treatment at 2.5, 5, and 10 mg/kg/day, respectively, for consecutive 45 days. All doses with a strength of 0.5, 1, and 2 mg/ml were freshly prepared in a solvent containing 0.5% w/v of CMC with the addition of 0.2% w/v of NaHCO_3_ to alkaline the gastric pH of the rats and prevent the degradation of esomeprazole and lansoprazole in acidic media [19]. The doses were administered orally using a gavage tube once daily in a fasting condition which further assisted in enhancing the gastric stability of both esomeprazole and lansoprazole.

### 2.4. Sample Collection

24 h post administration of last administered doses, the animals were anesthetized with diethyl ether and blood was withdrawn from each animal via cardiac puncture in plain vials. The serum was separated and stored at −20°C until the measurements of testosterone, FSH, and LH [20]. The anesthetized rats were sacrificed and the cauda epididymis of each rat was immediately removed for analysis of sperm parameters. The right and left testis were also removed for histological and oxidative stress biomarkers evaluations. The left testis of each animal was weighed, placed in phosphate buffer saline (PBS) of pH 7.4, and kept at −20°C until the analysis of oxidative stress biomarkers. The right testis of each animal was placed in a 10% formalin solution for histological analysis.

### 2.5. Sperm Analysis

The sperms were released from the separated cauda epididymis in a prewarmed petri dish carrying 1 ml PBS and allowed to disperse in the buffer for 1 min [21]. The sperm suspension obtained was then used for the analysis of sperm motility, sperm count, morphology, and viability.

### 2.6. Sperm Motility

A 10 *μ*l semen suspension was transferred on a slide (prewarmed at 37°C), and a computer-assisted semen analysis (CASA) System (Minitube®, Germany) with a phase-contrast microscope (Olympus Life Science®, Japan) was used to immediately assess the sperm sample at 40 x magnification [22]. About 500 sperms from minimum four different fields were examined in each sample for motility parameters. The data generated by the CASA system regarding motility parameters included sperm count, total and progressive motility, curvilinear velocity (VCL), straight-line velocity (VSL), average path velocity (VAP), and linearity (LIN) [23].

### 2.7. Sperm Morphology and Viability

A 10 *μ*l of semen suspension was stained with 10 *μ*l of 1% eosin stain. After 15 S, 20 *μ*l of 10% nigrosin stain was added and mixed together. A smear was formed by sliding another slide over it [24]. The air-dried slides were placed under a light microscope at 40 x magnification. A total of two hundred sperms were randomly analyzed to detect the morphological abnormalities and viability of sperms.

### 2.8. Reproductive Hormonal Analysis

The serum concentration of testosterone in ng/ml was obtained by following the directions supplied with the Access Testosterone assay kit (Elab Science, China) [25]. The detection range and sensitivity of the testosterone assay kit were 0.31–20 ng/ml and 0.17 ng/ml, respectively, with insignificant reactivity and interference with its analogues. On the other hand, the serum concentrations of FSH and LH were determined according to the directions provided with rat FSH (CAT No. E-EL-R0391) and (CAT No. LH E-EL-R0026) assay kits (Elab Science, China). The absorbance values of both FSH and LH were taken at 450 nm on a UV-Vis spectrophotometer and the concentrations were calculated by a standard equation. The detection range and sensitivity of the FSH assay kit were 3.13–300 ng/ml and 1.88 ng/ml, respectively, while the detection range for the LH assay kit was 1.56–10 MIU/ml and 0.94 MIU/ml with insignificant intra- and interassay variability. All samples were analyzed in triplicate.

### 2.9. Oxidative Stress Biomarkers Analysis

The testis tissues were isolated and weighed. Then, these tissues were homogenized in PBS (1 : 10 w/v) with a homogenizer followed by the centrifugation at 3000 rpm for 10 min. The supernatant fraction was separated and subsequently used for the analysis of superoxide dismutase (SOD), catalase (CAT), reduced glutathione (GSH), malondialdehyde (MDA), and NO. Moreover, the protein content in testis tissue homogenate was determined by the Lowry method [26].

### 2.10. MDA Level

MDA level is a widely used biomarker for the detection of lipid oxidation and is determined by TBA reagent [27]. For assessment of MDA level, 1 ml of sample supernatant was mixed with 3 ml of TBA reagent, heated for 0.5 h at 95°C, and then cooled in an ice bath. Following centrifugation at 3000 rpm for 10 min, the supernatant layer was removed to determine the absorbance at 532 nm using a UV-Vis spectrophotometer. The MDA values were expressed as nmol/ml of protein.

### 2.11. SOD Activity

Free radical scavenging SOD activity was determined by a method previously reported [28]. For measurement of SOD activity, 2.8 ml of PBS was transferred to 0.1 ml of tissue homogenate followed by mixing with 0.1 ml pyrogallol solution. Changes in absorbance were measured at 325 nm after every half minute for 5 min with a UV-Vis spectrophotometer. SOD activity was determined by using a regression equation and the result values were indicated as U/mg of protein.

### 2.12. GSH Level

For assessment of GSH level, 1 ml each of supernatant of testis homogenates and 10% TCA were added and mixed with 4 ml of PBS and 0.5 ml of DTNB reagent. The absorbance was recorded at 412 nm using a UV-Vis spectrophotometer. The values of GSH level were expressed as nmol/ml [29].

### 2.13. CAT Activity

It was determined by a previously established method [30]. For assessment of CAT, 5 *μ*l of supernatant and 1.95 mL of PBS were admixed with 1 mL of hydrogen peroxide (30 mM). Changes in absorbance were taken at 240 nm at 15 S interval for the 30 S with UV-Vis spectrophotometer. CAT activity was determined by the regression equation, and the result values were expressed as U/mg of protein.

### 2.14. NO Level

The level of NO was measured by mixing 1 ml of testis homogenate supernatant and 1 ml of Griess reagent and incubation for 10 min while the absorbance was determined at 540 nm using a UV-Vis spectrophotometer. NO level was estimated by the regression equation, and the result values were indicated as *μ*mol/mg of protein [31].

### 2.15. Histopathological Analysis

Right testis from animals was embedded in paraffin wax after being fixed in 10% formalin. Thin sections of 4 *μ*m of paraffin-embedded tissues were cut longitudinally and then stained with hematoxylin-eosin. The prepared slides were examined under a light microscope at 40x magnification, and microphotography was done for histopathological analysis [32]. These slides were evaluated for Johnsen Scoring [33]. Complete spermatogenesis was scored as 10. Several spermatids in seminiferous tubules were scored as 9 while score 8 was allocated to a few late spermatids with disrupted epithelium. Seminiferous tubules showing no late spermatids but early spermatids were allocated a score 7, whereas those with a few early spermatids and arrested spermatogenesis were scored 6. Johnsen scores of 5 and 4 were allocated to seminiferous tubules without spermatids but presenting many and a few spermatocytes, respectively. Seminiferous tubules which showed only spermatogonia and Sertoli cells were assigned Johnsen score 3 and 2, respectively. Johnsen score 1 was given to seminiferous tubules without any epithelial cells [33].

### 2.16. Statistical Analysis

The data were presented as mean ± standard deviation and analyzed by one-way ANOVA followed by Tuckey's post hoc test for multiple comparisons with the level of statistical significance set at *P* < 0.05, using GraphPad prism 8 (San Diego, CA) software. The statistical differences between the control and treatment groups as well as among the treatment groups were determined.

## 3. Results

Male rats exposed to esomeprazole and lansoprazole at a dose of 2.5, 5, and 10 mg/kg/day for 45 days showed that there was an insignificant change in diet and water uptake of all animal groups. There was no change in the general signs and symptoms such as respiration rate, body weight, physical activity, sleep, salivation, and reactivity to touch. The effects of esomeprazole and lansoprazole on sperm parameters including sperm count, motility, morphology and viability, reproductive hormones, and oxidative stress biomarkers are given here.

### 3.1. Sperm Motility

Animals treated with esomeprazole at 2.5, 5, and 10 mg/kg/day exhibited a significant (*P* < 0.0001) decline in total as well as progressive motility contrary to the control group. A significant reduction was noticed in sperm velocity parameters including VCL, VSL, and VAP in rats treated with esomeprazole at 2.5 mg/kg/day. However, there was a significant increase in LIN in animals treated with all dosage levels of esomeprazole in contrast to the control group as shown in Table 1. Insignificant differences were detected for VCL, VSL, and VAP in animals receiving esomeprazole 5 mg/kg/day in contrast to the control group. A significant reduction was observed in the VCL and VAP in animals treated with esomeprazole at 10 mg/kg/day contrary to the control group. The results obtained from epididymis sperm parameters posttreated with esomeprazole and lansoprazole are summarized in Table 1.

Animals treated with lansoprazole at all dosage levels showed a significant decline in total as well as progressive motility in contrast to the control group as shown in Table 1. There were insignificant differences in animals treated with lansoprazole at 2.5 and 10 mg/kg/day for VCL, VSL, VAP, and LIN in comparison to the control group. Treatment with lansoprazole at 5 mg/kg/day showed a significant reduction in sperm velocity parameters including VCL, VSL, and VAP than the control group. However, treatment with lansoprazole 5 mg/kg/day did not reduce LIN as compared to the normal control group.

### 3.2. Sperm Count and Testes Weight

A significant (*P* < 0.0001) reduction in sperm count was noticed in all animals treated with esomeprazole at 2.5, 5, and 10 mg/kg/day. However, treatment with esomeprazole at 10 mg/kg/day exhibited the most significant decline (28 ± 2 million/ml) in sperm count in comparison with the normal control group as shown in Table 2.

A significant (*P* < 0.0001) decline in sperm count was detected among all groups treated with lansoprazole at 2.5, 5, and 10 mg/kg/day. However, treatment with lansoprazole at 10 mg/kg/day showed the most pronounced decline (32 ± 2 million/ml) in sperm count in contrast to the control group. The results obtained from the sperm count of different rat groups after treatment with esomeprazole and lansoprazole are summarized in Table 2. Moreover, there was an insignificant difference in the testes weight among all groups. The effect of treatment with esomeprazole and lansoprazole on testes weight is shown in Table 2.

### 3.3. Sperm Viability and Morphology

**Figure 1:**
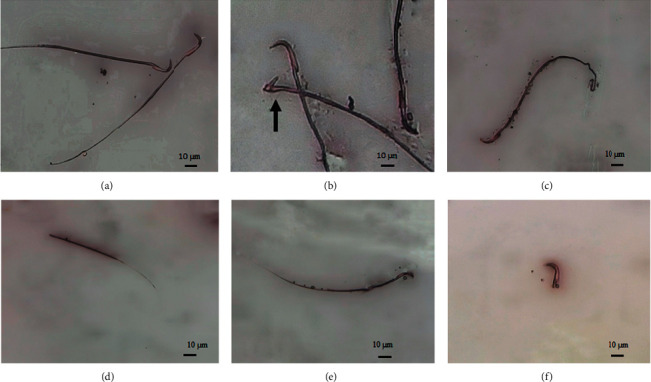
Sperm morphology of esomeprazole-treated rats at 2.5, 5, and 10 mg/kg/day (40 x). (a) Normal sperm, (b) bent neck sperm, (c) bent tail sperm, (d) headless tail sperm, (e) banana head sperm, and (f) detached head.

**Figure 2:**
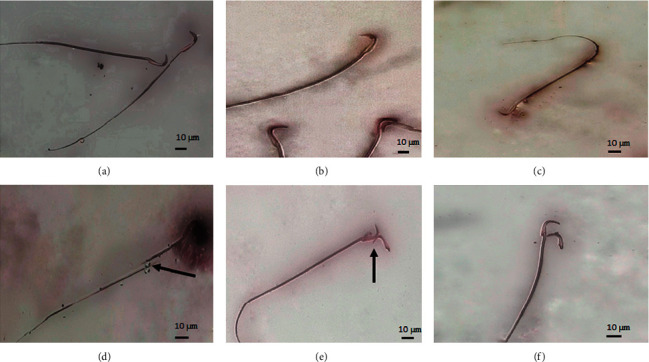
Sperm morphology in lansoprazole treated rats at 2.5, 5, and 10 mg/kg/day (40 x). (a) Normal sperm, (b) banana head sperm, (c) bent tail sperm, (d) amorphous head with a broken tail, (e) detached head, and (f) double head.

In animals treated with esomeprazole and lansoprazole, the dead sperms appeared with pink coloration of the head, while the live sperms appeared with a whitish or colorless head. Significant head and tail morphological abnormalities in animals treated with esomeprazole and lansoprazole at all dosage levels were observed and classified as bent neck, banana head, detached head, bent tail, and headless tail as shown in Figures 1 and 2.

Bent neck and tail sperm abnormalities were seen in animals treated with esomeprazole 2.5 mg/kg/day. Animals exposed to esomeprazole at 5 mg/kg/day exhibited detached head, bent neck, and tail abnormalities. Furthermore, banana head, headless tail, and bent tail abnormalities were noticed in animals receiving esomeprazole at 10 mg/kg/day in comparison to the normal control group as shown in Figure 1. Bent neck (34%) and bent tail (31%) were the most common abnormalities in esomeprazole-treated rats followed by banana head (26%) and headless tail (6%), respectively.

Detached head and banana head abnormalities were evident in animals receiving lansoprazole 2.5 mg/kg/day contrary to the control group. Treatment with lansoprazole at 5 mg/kg/day exhibited amorphous sperm head with a broken tail and bent tail abnormalities. Furthermore, double-headed and bent tail sperm abnormalities were noticed at lansoprazole at 10 mg/kg/day as shown in Figure 2. Bent neck (38%) and bent tail (33%) were the most common abnormalities in lansoprazole-treated rats followed by banana head (22%) and headless tail (7%), respectively.

### 3.4. Reproductive Hormones

The effects of esomeprazole and lansoprazole treatment on mean serum testosterone, FSH, and LH levels are summarized here.

### 3.5. LH Level

**Figure 3:**
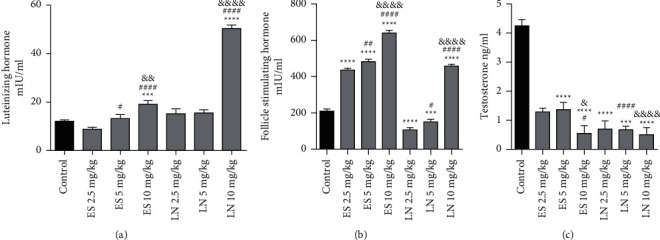
Effect of esomeprazole (ES) and lansoprazole (LN) treatment on sex hormones in male rats ^*∗∗∗*^ and ^*∗∗∗∗*^ showed significant results at *P* < 0.001 and *P* < 0.0001 contrary to control group. ^#^ and ^####^ showed significant results at *P* < 0.05 and 0.0001 on contrary to esomeprazole/lansoprazole 2.5 mg/kg/day. ^&^, ^&&^, and ^&&&&^ showed significant results at *P* < 0.05, 0.01, and 0.0001 on contrary to esomeprazole/lansoprazole 5 mg/kg/day.

Treatment with esomeprazole showed a significant (*P* < 0.001) rise in LH level at 10 mg/kg/day (19.05 ± 1.57 mIU/ml) as depicted in Figure 3. There was an insignificant difference in LH level of animals treated with esomeprazole at 2.5 mg/kg/day (8.80 ± 0.76 mIU/ml protein) and 5 mg/kg/day (13.16 ± 1.66 mIU/ml) on the contrary to the control group.

On the other hand, the LH level also significantly (*P* < 0.001) increased in animals exposed to lansoprazole at 10 mg/kg/day (50.36 ± 1.43 mIU/ml) on the contrary to the control group. No significant difference was seen on the LH level in animals exposed to lansoprazole at 2.5 mg/kg/day (15.17 ± 2.07 mIU/ml protein) and 5 mg/kg/day (15.54 ± 1.29 mIU/ml) in comparison with the control group. The effect of 45 days of treatment with esomeprazole and lansoprazole on LH level is shown in Figure 3.

### 3.6. FSH Level

A significant (*P* < 0.0001) rise in FSH level was noted in all animals treated with esomeprazole at all dosage levels in contrast to the control group. Treatment with esomeprazole at 10 mg/kg/day showed the most significant increase (640.40 ± 14.89 mIU /ml) in FSH level in comparison with the control group as presented in Figure 3.

**Figure 4:**
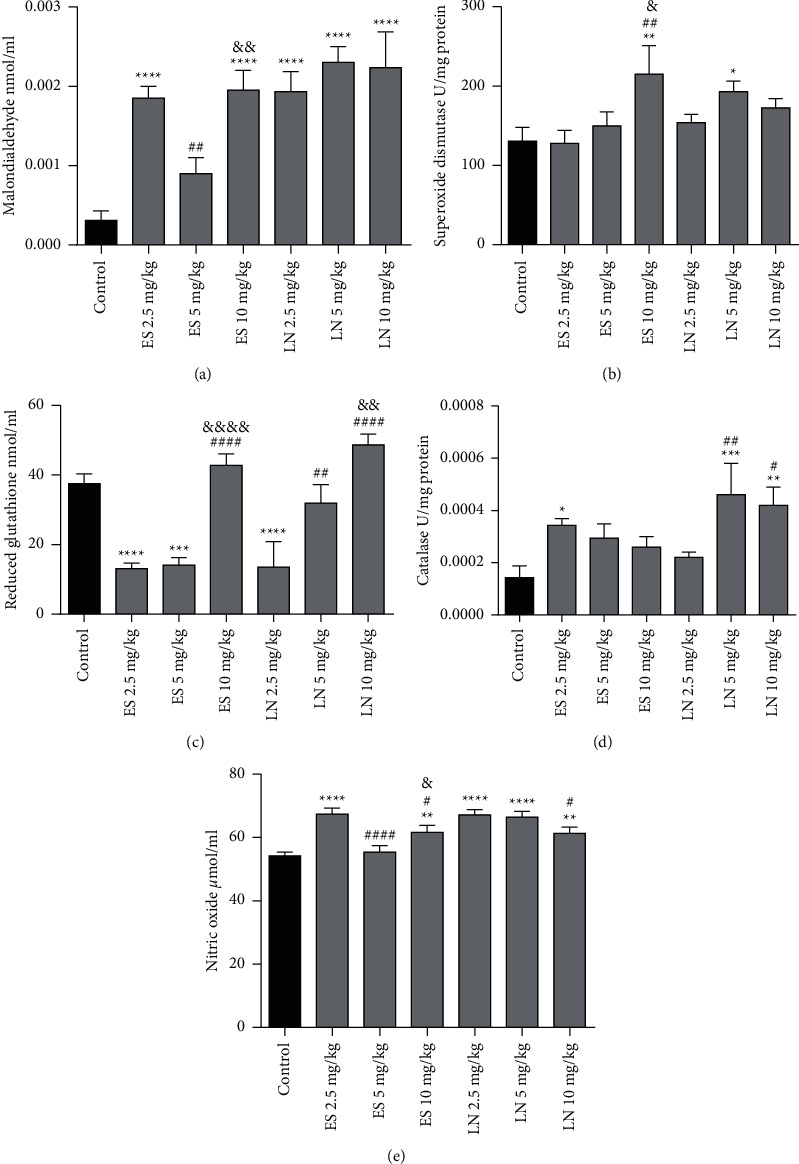
Effect of esomeprazole (ES) and lansoprazole (LN) treatment on oxidative stress biomarkers in rat testis. ^*∗*^, ^*∗∗*^, ^*∗∗∗*^, and ^*∗∗∗∗*^ showed significant results at *P* < 0.05, 0.01, 0.001, and 0.0001 on contrary to control group. ^#^, ^##^, and ^####^ showed significant results at *P* < 0.05, 0.01, and 0.0001 on contrary to esomeprazole/lansoprazole 2.5 mg/kg/day.^&^,^&&^, and ^&&&&^ showed significant results at *P* < 0.05, 0.01, and 0.0001 in contrary to esomeprazole/lansoprazole 5 mg/kg/day.

A significant (*P* < 0.0001) rise in FSH level was also detected in animals treated with lansoprazole at 10 mg/kg/day (458.40 ± 9.85 mIU/ml) in contrast to the control group as shown in Figure 4. However, FSH level significantly decreased in animals treated with lansoprazole at 2.5 mg/kg/day (106.40 ± 12.84 mIU /ml) and 5 mg/kg/day (150.40 ± 14.74 mIU /ml) in contrast to the control group (Figure 3).

### 3.7. Testosterone Level

It was observed that the testosterone level had reduced in all animals treated with esomeprazole at 2.5, 5, and 10 mg/kg/day in comparison to the control group. Treatment with esomeprazole at 10 mg/kg/day exhibited the most significant decline (0.55 ± 0.27 ng/ml) in testosterone level contrary to the control group as shown in Figure 3. A significant (*P* < 0.0001) reduction in testosterone level was also noticed in animals which received lansoprazole 2.5 mg/kg/day (0.70 ± 0.28 ng/ml) and 10 mg/kg/day (0.50 ± 0.34 ng/ml) in comparison to control group. Animals treated with lansoprazole at 5 mg/kg/day revealed a significant (*P* < 0.001) decrease in testosterone level (0.64 ± 0.28 ng/ml) in contrast to the control group as shown in Figure 3.

### 3.8. Oxidative Stress Biomarkers

After dissection, the left testicles from rats were removed and weighed. Rat testicular tissue (1 g) was homogenized in PBS to obtain the supernatant fraction that was used to measure biomarkers of oxidative stress. The overall effect of esomeprazole and lansoprazole treatment on the biomarkers of oxidative stress in testis is summarized here.

### 3.9. MDA Level

A significant (*P* < 0.0001) rise in MDA level in rat testis was noticed in esomeprazole-treated groups at 2.5 and 10 mg/kg/day in contrast to the control group. However, treatment with esomeprazole at 10 mg/kg/day showed the most significant (0.0019 ± 0.00025 nmol/ml) increase in MDA level contrary to the control group. An insignificant difference in the MDA level in animals treated with esomeprazole at 5 mg/kg/day was observed in comparison with the control group as presented in Figure 4.

MDA level also significantly (*P* < 0.0001) increased in rats exposed to lansoprazole at 2.5 mg/kg/day (0.0019 ± 0.00025 nmol/ml), 5 mg/kg/day (0.0023 ± 0.0002 nmol/ml), and 10 mg/kg/day (0.0022 ± 0.00045 nmol/ml) in contrary to the control group. However, lansoprazole-treated animals at 5 and 10 mg/kg/day showed the most significant increase in MDA level in contrast to control group. The effect of 45 days of treatment of lansoprazole on MDA level is demonstrated in Figure 4.

### 3.10. SOD Activity

SOD activity was significantly (*P* < 0.01) raised in esomeprazole-exposed animals at 10 mg/kg/day (215.32 ± 35.84 U/mg protein) in comparison with the control group. The rest of the groups did not show any significant difference, but a trend of increased SOD activity was observed contrary to the control group. Treatment with esomeprazole 2.5 mg/kg/day was insignificantly different from esomeprazole 5 mg/kg/day as presented in Figure 4. Furthermore, the SOD activity also significantly (*P* < 0.05) increased in lansoprazole-treated animals at 5 mg/kg/day (193.05 ± 13.47 U/mg protein) in contrast to the control group. Insignificant differences in SOD activity of animals treated with lansoprazole at 2.5 and 10 mg/kg/day were observed in comparison with the control group as presented in Figure 4.

### 3.11. GSH Level

The level of GSH was significantly reduced in animals that received esomeprazole 2.5 mg/kg/day (13.11 ± 1.59 nmol/ml) and 5 mg/kg/day (14.13 ± 2.16 nmol/ml) in contrast to the control group. There were insignificantly different GSH levels in animals that received esomeprazole 10 mg/kg/day (42.81 ± 3.29 nmol/ml) in comparison with the control group. The effect of 45 days of treatment with esomeprazole on GSH level in rat testis is shown in Figure 4.

In addition, the lansoprazole treatment also exhibited significant (*P* < 0.0001) reduction in GSH level at 2.5 mg/kg/day (13.52 ± 7.32 nmol/ml) in comparison with the control group as shown in Figure 4. There was an insignificant effect on the GSH level in animals treated with lansoprazole at 5 mg/kg/day (31.95 ± 5.31 nmol/ml) and 10 mg/kg/day (48.67 ± 3.10 nmol/ml) on the contrary to the control group (Figure 4).

### 3.12. CAT Activity

The CAT activity was significantly (*P* < 0.05) elevated in animals treated with esomeprazole 2.5 mg/kg/day (0.000343 ± 0.000025 U/mg protein) in comparison with the control group as shown in Figure 4. There was insignificantly different CAT activity in animals treated with esomeprazole 5 mg/kg/day (0.000293 ± 0.000055 U/mg protein) and 10 mg/kg/day (0.00026 ± 0.00004 U/mg protein) in contrast to the control group.

Moreover, CAT activity also significantly elevated in lansoprazole-treated animals at 5 mg/kg/day (0.00046 ± 0.00012 U/mg protein) and 10 mg/kg/day (0.00042 ± 0.00007 U/mg protein) in comparison with the control group. There was insignificantly different CAT activity in animals treated with lansoprazole at 2.5 mg/kg/day (0.00022 ± 0.00002 U/mg protein) in comparison with the control group. The effect of lansoprazole treatment on CAT activity in rat testes is displayed in Figure 4.

### 3.13. Nitric Oxide Level

The level of NO significantly increased in testicles of animals treated with esomeprazole at 2.5 mg/kg/day (67.28 ± 1.97 *μ*mol/ml) and 10 mg/kg/day (61.58 ± 2.20 *μ*mol/ml) as compared to control group. The insignificant effect of esomeprazole 5 mg/kg/day on nitric oxide level was observed in contrast to the control group as depicted in Figure 4.

Furthermore, the NO level was significantly elevated in all animals exposed to lansoprazole at 2.5 mg/kg/day (67.15 ± 1.59 *μ*mol/ml), 5 mg/kg/day (66.44 ± 1.82 *μ*mol/ml), and 10 mg/kg/day (61.27 ± 1.96 *μ*mol/ml) in contrast to control group as shown in Figure 4.

### 3.14. Histopathological Features

**Figure 5:**
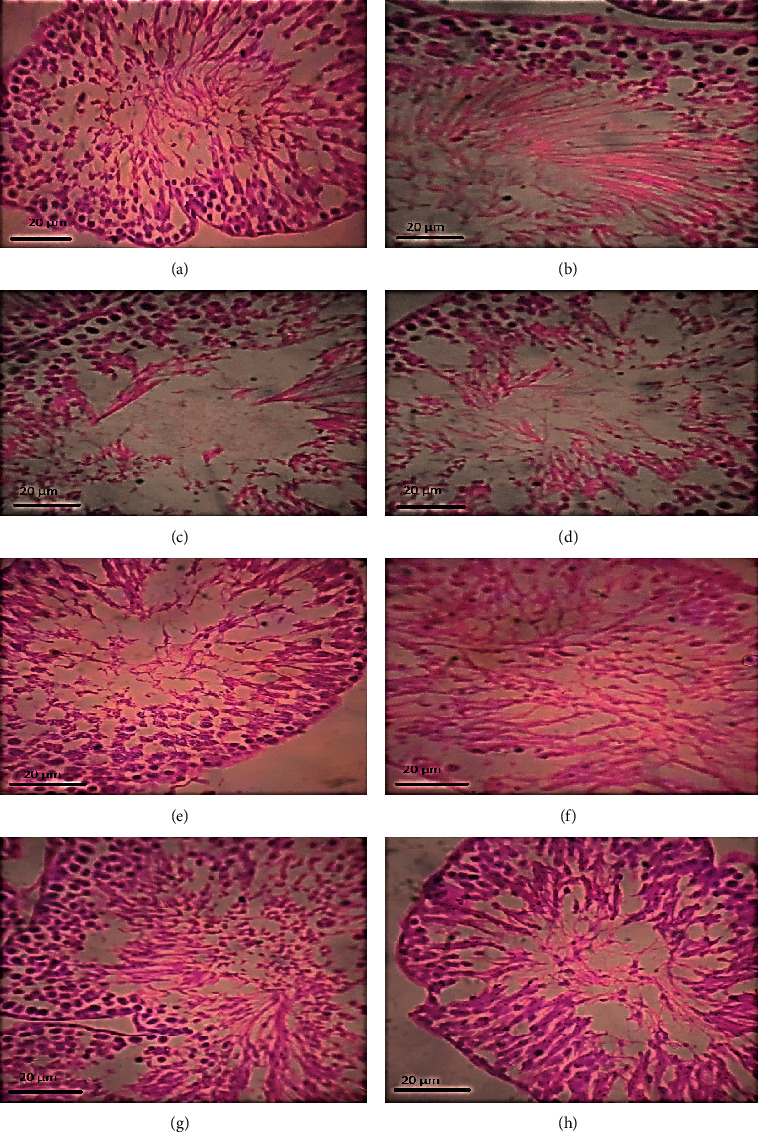
Photomicrograph of sections of seminiferous tubules and interstitial cells in esomeprazole (ES) and lansoprazole (LN) treated rats under magnification 100 x. (a) Control rats showed normal seminiferous tubules, (b) ES at 2.5 mg/kg/day showed oligospermia, vacuolization, and dilation/swelling of sperm cells, (c) ES at 5 mg/kg/day showed dilation of seminiferous tubules, (d) esomeprazole at 10 mg/kg/day showed swelling of Leydig cells, (e) control rats showed normal seminiferous tubules, (f) LN at 2.5 mg/kg/day showed oligospermia, vacuolization, dilation/swelling of sperm cells, and degenerated seminiferous tubules, (g) LN at 5 mg/kg/day showed dilation of seminiferous tubules, oligospermia dilation/swelling of sperm cells, vacuolization, necrosis, and swelling of Leydig cells, and (h) LN at 10 mg/kg/day showed vacuolization, dilation/swelling of sperm cells, and necrosis.

Different sections from the testicles were prepared by staining with hematoxylin-eosin stain and observed under a light microscope. Oligospermia, vacuolization, and dilation/swelling of sperm cells were detected in animals treated with esomeprazole at 2.5 mg/kg/day in contrast to control group as shown in Figure 5. On the other hand, oligospermia was seen in animals treated with lansoprazole at 2.5 and 5 mg/kg/day. Vacuolization and dilation/swelling of sperm cells were detected in all animals treated with lansoprazole at all dosage levels.

Furthermore, dilation of the seminiferous tubules and swelling of Leydig cells was noticed in lansoprazole-treated animals at 5 mg/kg/day. Animals treated with lansoprazole at 2.5 mg/kg/day showed degeneration in seminiferous tubules. Animals treated with lansoprazole at 5 and 10 mg/kg/day also exhibited necrosis in the seminiferous tubules (Figure 5).

**Figure 6:**
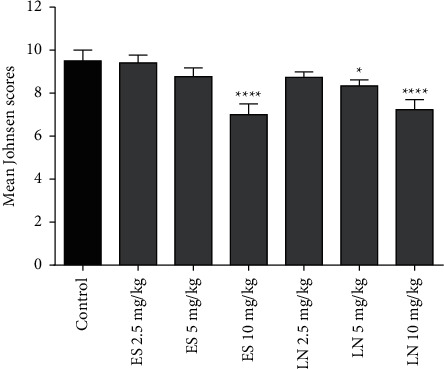
Histopathological scoring of testes from esomeprazole (ES) and lansoprazole (LN) treated rats. ^*∗*^*P* < 0.05 and ^*∗∗∗∗*^*P* < 0.0001 showed a significant difference from the normal control group.

It was demonstrated by quantitative histopathological scoring that there was an insignificant reduction of scores in animals treated with esomeprazole 2.5 and 5 mg/kg/day, and lansoprazole 2.5 mg/kg/day in comparison to normal control. Animals treated with esomeprazole 10 mg/kg/day, and lansoprazole 5 or 10 mg/kg/day showed significant histopathological anomalies as depicted by lower Johnsen scores as compared to the normal control group. The effects of treatment with esomeprazole and lansoprazole on Johnsen scores in rats are demonstrated in Figure 6.

## 4. Discussion

The prevalence of male infertility has an unpleasant impact on the quality of life [34]. Several studies have reported a strong correlation between male infertility and drug-related undesirable effects [35]. In the current study, the harmful effects of PPIs on sperm function and male fertility were evaluated. The most commonly used PPIs, esomeprazole and lansoprazole, were orally administered at 2.5, 5, and 10 mg/kg/day dosage to male Wistar rats for 45 consecutive days to demonstrate their effects on sperm parameters, sex hormones, oxidative stress, and testicular histology.

The sperm parameters including sperm count and motility are important predictors of male infertility and are responsible for fertilization. The current study indicated a significant reduction in total as well as progressive sperm motility of animals exposed to esomeprazole and lansoprazole at all dose levels. CASA-based sperm velocity analysis also showed a significant decline in VSL, VCL, VAP, and LIN parameters in animals exposed to esomeprazole. Increased sperm velocity (VSL, VCL, VAP) and decreased linearity (LIN = VSL/VCL) are related to hyperactivated motility, thus, important for initiation of capacitation and acrosomal reaction [23]. The underlying mechanism for reduced sperm motility could be the inhibitory action of esomeprazole and lansoprazole on the choline acetyl-transferase enzyme responsible for the biosynthesis of acetylcholine (Ach). Previous in vitro study indicated that the reduction in sperm motility was due to reduced synthesis of Ach, a chemotactic agent for sperm motility [36]. There was an insignificant decrease in sperm motility parameters at low and high doses of esomeprazole in the current study. It was found that esomeprazole had shown a significant decline in all sperm motility parameters on subchronic exposure, which was in accordance with a previous ex vivo study of PPIs on human sperm motility [37].

In the present study, the sperm count in rats significantly declined post administration of esomeprazole and lansoprazole at all dose levels. It was revealed that both esomeprazole and lansoprazole had exhibited the most significant decline in sperm count at the maximum administered dose of 10 mg/kg/day. The crucial role of PPIs in reducing sperm count was the result of excessive production of nitric oxide [24]. A high level of nitric oxide caused oxidative stress leading to sperm damage and infertility [38]. The other mechanism involved in reduced sperm count could be the direct effect of PPIs in increasing the gastric pH, resulting in reduced uptake of water-soluble vitamins and other nutrients essential for spermatogenesis [39].

The presence of any morphological abnormality in sperms adversely affects sperm quality and fertility [40]. In the current study, significant morphological abnormalities were seen in animals treated with esomeprazole and lansoprazole at all dose levels. Possibly, the induction of head and tail abnormalities of sperm could be due to the vulnerability of spermatozoa to oxidative stress-induced DNA damage which agrees with previous in vivo study on male infertility [41]. The ROS also target the polyunsaturated fatty acids for detrimental peroxidation leading to decreased membrane permeability and increased structural defects of sperms [10].

Altered level of serum testosterone and gonadotropins (FSH and LH) is often associated with abnormal spermatogenesis. It is well documented that an adequate level of testosterone plays a pivotal role in sperm cell development and spermatogenesis [42]. Studies also indicated that serum FSH and LH levels have an inverse relation with sperm concentration. In the current study, esomeprazole and lansoprazole treatment indicated a significant decline in serum testosterone level at all dose levels which led to impaired spermatogenesis. Both esomeprazole and lansoprazole showed the most significant decline in serum testosterone levels at the maximum concentration of 10 mg/kg/day. Conversely, this study also indicated a significant increase in serum gonadotropins (FSH and LH) in animals exposed to esomeprazole and lansoprazole at the maximum concentration of 10 mg/kg/day. It is further known that the testosterone synthesis by Leydig cells is dependent upon the actively functioning mitochondria where the ATP generation occurs as a consequence of pH differential potential due to proton gradient generated by the pumping of protons from matrix to an inter-membranous region of mitochondria through proton pumps. The decreased level in testosterone of animals exposed to PPIs in the current study can be attributed to the inhibition of proton pumps by esomeprazole and lansoprazole [43].

Reduction in serum testosterone and an increase in serum FSH and LH were considered useful indicators of impaired spermatogenesis, testicular damage, and oligozoospermia [44]. These outcomes are suggested to be caused by the esomeprazole- and lansoprazole-induced hepatic metabolism of testosterone resulting in reduced levels of serum testosterone [45]. Simultaneously, the serum level of FSH and LH were raised due to disturbance in the negative feedback mechanism in the HPG axis [46]. Intracellular pH of Sertoli cells plays a pivotal role in spermatogenesis modulation which is carried out by various proton transporters and co-transporters. This intracellular pH regulates the cellular metabolism and differentiation of Sertoli cells. Inhibition of these proton pump cells by PPIs could be one of the mechanisms for the reduction in spermatogenesis of Sertoli cells [47].

Oxidative stress is an imbalance between the generation of reactive ROS and the antioxidant ability of cells [48]. The oxidative stress in testicles is strongly correlated with male infertility. In a previous study, elevated levels of ROS had negatively impacted the normal sperm function by inducing oxidative stress [49]. It is well known that sperms are vulnerable to ROS because the cell membrane of sperm comprises a high level of polyunsaturated fatty acids while the reduced amount of ROS neutralizing enzyme is present in the cytoplasm [48]. Results of the current study showed that esomeprazole and lansoprazole treatment caused a significant decrease in CAT activity (10 mg/kg/day) and GSH level (2.5 mg/kg/day). In contrast, both esomeprazole and lansoprazole treatment exhibited a significant increase in MDA (at 10 mg/kg/day) and NO level (at 2.5 mg/kg/day).

Furthermore, the SOD activity was significantly raised in animals exposed to esomeprazole treatment (10 mg/kg/day) and lansoprazole treatment (5 mg/kg/day). An elevated level of MDA in esomeprazole and lansoprazole treatment groups was an indicator of lipid peroxidation, which is in accordance with the previous study [50]. It was also reported that oxidative stress was related to significantly decrease GSH levels and increased activity of SOD to cope with an increased ROS. Additionally, there are many supportive studies indicating that oxidative stress is coupled with an elevated level of NO [51]. Aside from a reduced generation of ATP, elevated NO causes sperm cell apoptosis, inhibition of Leydig cell steroidogenesis, decreased sperm viability, and altered morphology via peroxidative damage to the sperm membrane [14]. In another study, PPIs were also shown to induce oxidative stress in human melanoma cells which support the current findings [52]. It is established that the FSH acts on Sertoli cells to induce spermatogenesis, in addition to purinergic signaling through adenosine and ATP. Oxidative stress alters purinergic signaling and activities of cholinesterase, chymotrypsin, and other enzymes through the generation of oxidative metabolites and thus adversely impacts Sertoli cell functioning even in the presence of FSH [53]. Moreover, reduced spermatogenesis by Sertoli cells even in the presence of testosterone and FSH support indicates idiopathic infertility [54].

In this study, esomeprazole- and lansoprazole-treated rats exhibited vacuolization, oligospermia, degeneration of seminiferous tubules, swelling of Leydig cells, and necrosis in seminiferous tubules. This testicular damage and reduced sperm cells could be due to the elevated level of ROS and decreased level of serum testosterone [55]. The possible mechanisms of PPIs induced deleterious effects on male fertility include inhibition of choline acetyltransferase, inhibition of vas deferens contractility, and production of NO [37].

## 5. Conclusion

In conclusion, 45 days of administration of esomeprazole and lansoprazole induced the reproductive adverse effects in male rats. Subchronic exposure to these agents had reduced male fertility indicated by the impaired sperm quality, abnormal sperm morphology, reduced sperm count, and motility. Reproductive toxicity of PPIs was mediated through an elevation in NO, FSH, and LH levels, and a reduction in serum testosterone. Moreover, these drugs induced oxidative stress in testicular tissues through reduction of CAT activity and GSH level, and increased lipid peroxidation. Considering these findings and the widespread use of PPIs, further studies are needed specifically in clinical settings to monitor sperm parameters and reproductive hormone levels in patients using PPIs.

## Figures and Tables

**Table 1:** Effect of treatment with esomeprazole (ES) and lansoprazole (LN) on sperm motility in male rats.

Groups	Total motility (%)	Progressive motility (%)	Curvilinear velocity (*μ*m/s)	Straight line velocity (*μ*m/s)	Average path velocity (*μ*m/s)	Linearity (%)
Control	77.60 ± 0.83	63.73 ± 1.47	151.26 ± 23.84	110.04 ± 15.76	115.27 ± 15.87	0.73 ± 0.01
ES 2.5 mg/kg/day	48.67 ± 2.22^*∗∗∗∗*^	29.16 ± 1.41^*∗∗∗∗*^	79.40 ± 10.05^*∗∗∗∗*^	63.88 ± 9.90^*∗∗∗*^	66.62 ± 10.19^*∗∗∗*^	0.80 ± 0.02^*∗∗∗*^
ES 5 mg/kg/day	53.14 ± 1.89^*∗∗∗∗*^	40.53 ± 0.22^*∗∗∗∗*^	124.72 ± 2.53	99.10 ± 5.26	102.66 ± 7.23	0.79 ± 0.01^*∗∗∗*^
ES 10 mg/kg/day	50.27 ± 1.99^*∗∗∗∗*^	41.60 ± 0.34^*∗∗∗∗*^	110.72 ± 3.53^*∗*^	85.92 ± 2.42	89.49 ± 2.67^*∗*^	0.77 ± 0.005^*∗*^
LN 2.5 mg/kg/day	60.56 ± 1.74^*∗∗∗∗*^	49.31 ± 1.51^*∗∗∗∗*^	135.32 ± 5.26	101.91 ± 5.71	105.72 ± 5.78	0.75 ± 0.01
LN 5 mg/kg/day	62.19 ± 2.06^*∗∗∗∗*^	43.25 ± 1.17^*∗∗∗∗*^	96.78 ± 12.77^*∗∗*^	75.18 ± 12.07^*∗∗*^	79.04 ± 11.79^*∗∗*^	0.77 ± 0.02^*∗*^
LN 10 mg/kg/day	61.20 ± 2.38^*∗∗∗*^	47.58 ± 2.56^*∗∗∗∗*^	121.86 ± 19.80	90.93 ± 15.38	94.98 ± 15.61	0.74 ± 0.005

Results are shown as mean ± standard deviation. ^*∗*^, ^*∗∗*^, ^*∗∗∗*^, and ^*∗∗∗∗*^ showed significant results at *P* < 0.05, 0.01, 0.001, and 0.0001 in contrast to the normal control group.

**Table 2:** Effect of treatment with esomeprazole (ES) and lansoprazole (LN) on sperm count and testes weight in male rats.

Treatments	Sperm count (millions/ml)	Testes weight (g)
Normal control	74 ± 2	0.930 ± 0.172
ES 2.5 mg/kg/day	46 ± 3^*∗∗∗∗*^	0.853 ± 0.170
ES 5 mg/kg/day	42 ± 3^*∗∗∗∗*^	0.863 ± 0.134
ES 10 mg/kg/day	28 ± 2^*∗∗∗∗*^	0.856 ± 0.152
LN 2.5 mg/kg/day	45 ± 2^*∗∗∗∗*^	0.894 ± 0.162
LN 5 mg/kg/day	40 ± 2^*∗∗∗∗*^	0.882 ± 0.133
LN 10 mg/kg/day	32 ± 2^*∗∗∗∗*^	0.786 ± 0.146

Results are shown as mean ± standard deviation. ^*∗∗∗∗*^*P* < 0.0001 showed statistical difference in contrast to the normal control group.

## Data Availability

All data are included in the manuscript.

## References

[1] Monteiro C., Marques P. I., Cavadas B. (2018). Characterization of microbiota in male infertility cases uncovers differences in seminal hyperviscosity and oligoasthenoteratozoospermia possibly correlated with increased prevalence of infectious bacteria. *Ameriacn Jounral Reproductive Immunology*.

[2] Ajayi A. F., Akhigbe R. E. (2020). The physiology of male reproduction: Impact of drugs and their abuse on male fertility. *Andrologia*.

[3] Babakhanzadeh E., Nazari M., Ghasemifar S., Khodadadian A. (2020). Some of the factors involved in male infertility: a prospective review. *International Journal of General Medicine*.

[4] Ji H., Miao M., Liang H. (2018). Exposure of environmental bisphenol A in relation to routine sperm parameters and sperm movement characteristics among fertile men. *Scientific Reports*.

[5] Kumar N., Singh A. K. (2015). Trends of male factor infertility, an important cause of infertility: a review of literature. *Journal of Human Reproductive Sciences*.

[6] Ernesto J. I., Weigel Muñoz M., Battistone M. A. (2015). CRISP1 as a novel catsper regulator that modulates sperm motility and orientation during fertilization. *Journal of Cell Biology*.

[7] Nowicka-Bauer K., Lepczynski A., Ozgo M. (2018). Sperm mitochondrial dysfunction and oxidative stress as possible reasons for isolated asthenozoospermia. *Journal of Physiology and Pharmacology*.

[8] Xu X., Sha Y.-W., Mei L.-B. (2018). A familial study of twins with severe asthenozoospermia identified a homozygous SPAG17 mutation by whole-exome sequencing. *Clinical Genetics*.

[9] Agarwal A., Tvrda E., Sharma R. (2014). Relationship amongst teratozoospermia, seminal oxidative stress and male infertility. *Reproductive Biology and Endocrinology*.

[10] Colagar A. H., Karimi F., Jorsaraei S. G. A. (2013). Correlation of sperm parameters with semen lipid peroxidation and total antioxidants levels in astheno-and oligoasheno-teratospermic men. *Iran Red Crescent Medical Journal*.

[11] Dwyer A. A., Quinton R. (2019). Anatomy and physiology of the hypothalamic-pituitary-gonadal (HPG) axis. *Advanced Practice in Endocrinology Nursing*.

[12] Basaria S. (2014). Male hypogonadism. *Lancet*.

[13] Kolesnikova L. I., Kolesnikov S. I., Kurashova N. A., Bairova T. A. (2015). Causes and factors of male infertility. *Vestnik Rossiiskoi Akademeii Meditsinskikh Nauk*.

[14] Doshi S. B., Khullar K., Sharma R. K., Agarwal A. (2012). Role of reactive nitrogen species in male infertility. *Reproductive Biology and Endocrinology*.

[15] Bisht S., Faiq M., Tolahunase M., Dada R. (2017). Oxidative stress and male infertility. *Nature Reviews*.

[16] Nehra A. K., Alexander J. A., Loftus C. G., Nehra V. (2018). Proton pump inhibitors: review of emerging concerns. *Mayo Clinic Proceedings*.

[17] Clouston S. A., Shapira O., Kotov R. (2017). Proton pump inhibitors and the risk of severe cognitive impairment: The role of posttraumatic stress disorder. *Alzheimers Dement*.

[18] Escoffier J., Arnaud B., Kaba M. (2020). Pantoprazole, a proton-pump inhibitor, impairs human sperm motility and capacitation in vitro. *Andrology*.

[19] Van Nguyen H., Baek N., Lee B.-J. (2017). Enhanced gastric stability of esomeprazole by molecular interaction and modulation of microenvironmental pH with alkalizers in solid dispersion. *International Journal of Pharmaceutics*.

[20] Jaffat H. S., Obaid F. N. (2018). Determination of total antioxidant capacity, LH, FSH and testosterone in serum of male albino rats which orally given by finasteride (prostacare). *Journal of Pharmaceutical Sciences ans Research*.

[21] Ilgin S., Kilic G., Baysal M. (2017). Citalopram induces reproductive toxicity in male rats. *Birth Defects Research*.

[22] Morakinyo A., Iranloye B., Daramola A., Adegoke O., Adegoke O. A. (2011). Antifertility effect of calcium channel blockers on male rats: association with oxidative stress. *Advances in medical Sciences*.

[23] Adamkovicova M., Toman R., Martiniakova M. (2016). Sperm motility and morphology changes in rats exposed to cadmium and diazinon. *Reproductive Biology and Endocrinology*.

[24] Banihani S. A., Khasawneh F. H. (2018). Effect of lansoprazole on human sperm motility, sperm viability, seminal nitric oxide production, and seminal calcium chelation. *Research in Pharmaceutical Sciences*.

[25] Jahan S., Munawar A., Razak S. (2018). Ameliorative effects of rutin against cisplatin-induced reproductive toxicity in male rats. *BMC Urology*.

[26] Zulfqar F., Akhtar M. F., Saleem A., Akhtar B., Sharif A., Saleem U. (2020). Chemical characterization, antioxidant evaluation, and antidiabetic potential of *Pinus gerardiana* (pine nuts) extracts. *Journal of Food Biochemistry*.

[27] Akhtar M. F., Khan K., Saleem A., Baig M. M. F. A., Rasul A., Abdel-Daim M. M. (2021). Chemical characterization and anti-arthritic appraisal of *Monotheca buxifolia* methanolic extract in complete freund’s adjuvant-induced arthritis in wistar rats. *Inflammopharmacology*.

[28] Akhtar M. F., Raza S. A., Saleem A. (2021). Appraisal of anti-arthritic and anti-inflammatory potential of folkloric medicinal plant peganum harmala. *Endocrine, Metabolic & Immune Disorders Drug Targets*.

[29] Akhtar M. F., Younas S., Saleem A. (2021). Maternotoxicity and fetotoxicity in *Rattus norvegicusRattus norvegicus* albinus exposed to tramadol during the late phase of pregnancy. *Birth Defects Research*.

[30] Akhtar M. F., Zubair S., Saleem A., Alsharif K. F., Abdel-Daim M. M. (2021). Comparison of individual and combination treatments with naproxen, prednisolone and hydroxychloroquine to treat complete freund’s adjuvant induced arthritis. *Inflammopharmacology*.

[31] ElBeltagi H. S., Ahmed M. (2016). Assessment the protective role of quercetin on acrylamide-induced oxidative stress in rats. *Journal of Food Biochemistry*.

[32] Saleem A., Akhtar M. F., Latif A. (2021). Chemical characterisation and appraisal of antidiabetic potential of terminalia citrina extract in streptozotocin induced hyperglycaemia in wistar rats. *Archives of Physiology and Biochemistry*.

[33] Yari A., Asadi M. H., Bahadoran H., Dashtnavard H., Imani H., Naghii M. R. (2010). Cadmium toxicity in spermatogenesis and protective effects of L-carnitine in adult male rats. *Biological Trace Element Research*.

[34] Ilacqua A., Izzo G., Emerenziani G. P., Baldari C., Aversa A. (2018). Lifestyle and fertility: the influence of stress and quality of life on male fertility. *Reproductive Biology and Endocrinology*.

[35] Olayemi F. (2010). A review on some causes of male infertility. *African Journal of Biotechnology*.

[36] Kumar A., Kumar R., Flanagan J., Långström B., Björndahl L., Darreh-Shori T. (2020). Esomeprazole reduces sperm motility index by targeting the spermic cholinergic machinery: A mechanistic study for the association between use of proton pump inhibitors and reduced sperm motility index. *Biochemical Pharmacology*.

[37] Kumar R., Kumar A., Nordberg A., Långström B., Darreh-Shori T. (2020). Proton pump inhibitors act with unprecedented potencies as inhibitors of the acetylcholine biosynthesizing enzyme—a plausible missing link for their association with incidence of dementia. *Alzheimer’s & Dementia*.

[38] Ramya T., Misro M. M., Sinha D., Nandan D., Mithal S. (2011). Altered levels of seminal nitric oxide, nitric oxide synthase, and enzymatic antioxidants and their association with sperm function in infertile subjects. *Fertility & sterility*.

[39] Huijgen N. A., de Ridder M. A., Verhamme K. M. (2016). Are proton-pump inhibitors harmful for the semen quality of men in couples who are planning pregnancy?. *Fertility and Sterility*.

[40] Varshini J., Srinag B., Kalthur G. (2012). Poor sperm quality and advancing age are associated with increased sperm DNA damage in infertile men. *Andrologia*.

[41] Hosen M. B., Islam M. R., Begum F., Kabir Y., Howlader M. Z. H. (2015). Oxidative stress induced sperm DNA damage, a possible reason for male infertility. *Iranian Journal of Reproductive Medicine*.

[42] Soliman M. E., Mahmoud B. L., Kefafy M. A., Yassien R. I., El-Roghy E. S. A. (2017). Effect of antidepressant drug (fluoxetine) on the testes of adult male albino rats and the possible protective role of omega-3. *Menoufia Medical Journal*.

[43] Allen J. A., Shankara T., Janus P. (2006). Energized, polarized, and actively respiring mitochondria are required for acute leydig cell steroidogenesis. *Endocrinology*.

[44] Walker W. H. (2011). Testosterone signaling and the regulation of spermatogenesis. *Spermatogenesis*.

[45] Thanoon I. A.-J., Mahmood A. Q. (2011). Effect of omeprazole on reproductive hormonal levels and sexual function in male patients with peptic ulcer disease. *Al-Qadisiah Medical Journal*.

[46] Babu S. R., Sadhnani M., Swarna M., Padmavathi P., Reddy P. P. (2004). Evaluation of FSH, LH and testosterone levels in different subgroups of infertile males. *Indian Journal of Clinical Biochemistry*.

[47] Oliveira P., Sousa M., Barros A., Moura T., Rebelo da Costa A. (2009). Membrane transporters and cytoplasmatic pH regulation on bovine Sertoli cells. *Journal of Membrane Biology*.

[48] Migdal C., Serres M. (2011). Reactive oxygen species and oxidative stress. *Medicine Sciences*.

[49] Tremellen K. (2008). Oxidative stress and male infertility—a clinical perspective. *Human Reproductive Update*.

[50] Tsikas D. (2017). Assessment of lipid peroxidation by measuring malondialdehyde (MDA) and relatives in biological samples: analytical and biological challenges. *Analytical Biochemistry*.

[51] Förstermann U., Xia N., Li H. (2017). Roles of vascular oxidative stress and nitric oxide in the pathogenesis of atherosclerosis. *Curculation Research*.

[52] Marino M., Fais S., Djavaheri-Mergny M. (2010). Proton pump inhibition induces autophagy as a survival mechanism following oxidative stress in human melanoma cells. *Cell Death & Disease*.

[53] Erukainure O. L., Atolani O., Banerjee P. (2021). Oxidative testicular injury: effect of l-leucine on redox, cholinergic and purinergic dysfunctions, and dysregulated metabolic pathways. *Amino Acids*.

[54] Basu S., Arya S. P., Usmani A. (2018). Defective Wnt3 expression by testicular Sertoli cells compromise male fertility. *Cell and Tissue Research*.

[55] de Siqueira Bringel S., de Amorim Júnior A. A., Amorim M. J. A. A. L. (2013). Endocrine and testicular changes induced by olanzapine in adult wistar rats. *Journal of Applied Toxicology*.

